# Weekly Treatment of 2-Hydroxypropyl-β-cyclodextrin Improves Intracellular Cholesterol Levels in LDL Receptor Knockout Mice

**DOI:** 10.3390/ijms160921056

**Published:** 2015-09-02

**Authors:** Sofie M. A. Walenbergh, Tom Houben, Tim Hendrikx, Mike L. J. Jeurissen, Patrick J. van Gorp, Nathalie Vaes, Steven W. M. Olde Damink, Fons Verheyen, Ger H. Koek, Dieter Lütjohann, Alena Grebe, Eicke Latz, Ronit Shiri-Sverdlov

**Affiliations:** 1 Department of Molecular Genetics, School of Nutrition and Translational Research in Metabolism (NUTRIM), Maastricht University, Maastricht 6229ER, The Netherlands; E-Mails: s.walenbergh@maastrichtuniversity.nl (S.M.A.W.); tom.houben@maastrichtuniversity.nl (To.H.); t.hendrikx@maastrichtuniversity.nl (Ti.H.); m.jeurissen@maastrichtuniversity.nl (M.L.J.J.); p.vangorp@maastrichtuniversity.nl (P.J.G.); n.vaes@maastrichtuniversity.nl (N.V.); 2 Department of General Surgery, Maastricht University, Maastricht 6229ER, The Netherlands; E-Mail: steven.oldedamink@maastrichtuniversity.nl; 3 Department of HPB and Liver Transplantation Surgery, Royal Free Hospital, University College London, London NW3 2PF, UK; 4 Department of Molecular Cell Biology and Electron Microscopy, Maastricht University, Maastricht 6229ER, The Netherlands; E-Mail: f.verheyen@maastrichtuniversity.nl; 5 Department of Internal Medicine, Division of Gastroenterology and Hepatology, Maastricht University Medical Center (MUMC), Maastricht 6202AZ, The Netherlands; E-Mail: gh.koek@mumc.nl; 6 Institute of Clinical Chemistry and Clinical Pharmacology, University of Bonn, Bonn D-53105, Germany; E-Mail: dieter.luetjohann@ukb.uni-bonn.de; 7 Institute of Innate Immunity, University Hospital, University of Bonn, Bonn D-53127, Germany; E-Mails: alena.grebe@uni-bonn.de (A.G.); eicke.latz@umassmed.edu (E.L.); 8 German Center for Neurodegenerative Diseases (DZNE), Bonn D-53127, Germany; 9 Division of Infectious Diseases and Immunology, University of Massachusetts Medical School, Worcester, MA 01605, USA

**Keywords:** NAFLD, metabolic syndrome, cyclodextrin, electron microscopy, lysosomes

## Abstract

Recently, the importance of lysosomes in the context of the metabolic syndrome has received increased attention. Increased lysosomal cholesterol storage and cholesterol crystallization inside macrophages have been linked to several metabolic diseases, such as atherosclerosis and non-alcoholic fatty liver disease (NAFLD). Two-hydroxypropyl-β-cyclodextrin (HP-B-CD) is able to redirect lysosomal cholesterol to the cytoplasm in Niemann-Pick type C1 disease, a lysosomal storage disorder. We hypothesize that HP-B-CD ameliorates liver cholesterol and intracellular cholesterol levels inside Kupffer cells (KCs). Hyperlipidemic low-density lipoprotein receptor knockout (*Ldlr^−/−^*) mice were given weekly, subcutaneous injections with HP-B-CD or control PBS. In contrast to control injections, hyperlipidemic mice treated with HP-B-CD demonstrated a shift in intracellular cholesterol distribution towards cytoplasmic cholesteryl ester (CE) storage and a decrease in cholesterol crystallization inside KCs. Compared to untreated hyperlipidemic mice, the foamy KC appearance and liver cholesterol remained similar upon HP-B-CD administration, while hepatic campesterol and 7α-hydroxycholesterol levels were back increased. Thus, HP-B-CD could be a useful tool to improve intracellular cholesterol levels in the context of the metabolic syndrome, possibly through modulation of phyto- and oxysterols, and should be tested in the future. Additionally, these data underline the existence of a shared etiology between lysosomal storage diseases and NAFLD.

## 1. Introduction

Non-alcoholic fatty liver disease (NAFLD) describes several stages of liver disease characterized by no or little alcohol use, and is currently viewed as the precursor of the metabolic syndrome [1]. Initially, the excessive buildup of fat inside the liver, also referred to as steatosis, is a benign and reversible condition. However, later stages of NAFLD are characterized by liver inflammation, the formation of irreversible scar tissue (fibrosis-cirrhosis) and severe end-stage liver disease [2]. Currently, the prevalence of NAFLD is estimated to grow as a direct result of the global obesity epidemic [3]. Better insights into the mechanisms that cause NAFLD are required in order to develop novel therapeutic interventions.

Under healthy circumstances, lipoproteins are endocytosed by macrophages and initially directed to the endolysosomal compartment where further processing will take place. Subsequently, cholesterol is transferred from the lysosomes to the cytoplasm. Interestingly, previous studies from our group revealed that during hyperlipidemic conditions in mice, such as NAFLD, cholesterol is not transported into the cytoplasm, but rather accumulates inside lysosomes of the Kupffer cells (KCs). In addition to a resistance of cholesterol efflux from the lysosome, we observed increased cholesterol crystals in the livers of these mice [4,5]. These cholesterol crystals are so-called cholesterol deposits, formed upon excessive cholesterol uptake. Similar to our data, lysosomal cholesterol storage and cholesterol crystallization inside macrophages was also observed during atherosclerosis [6]. Therefore, the suggestion was raised that both these metabolic diseases share disease mechanisms and could be referred to as acquired lysosomal storage disorders [7,8]. A classical lysosomal storage disorder, such as Niemann-Pick type C (NPC) disease, is caused by a mutation in either the *Npc1* or *Npc2* gene, which encodes for a key protein that is responsible for cholesterol transport from the lysosomes to the cytoplasm. As a result, NPC disease patients demonstrate progressive accumulation of cholesterol inside lysosomes that severely damages almost all organs, leading to neurological disease, liver dysfunction and eventually premature death [9]. Notably, increased lysosomal cholesterol accumulation in *Npc1^−/−^* mice could be reversed by the administration of two-hydroxypropyl-β-cyclodextrin (HP-B-CD) and normalized the cholesterol metabolism in nearly every organ of the body [10,11,12,13,14]. Thus far, the effect of HP-B-CD on the cholesterol metabolism during NAFLD has never been studied.

The aim of the current study was to investigate whether HP-B-CD treatment is able to modify the cholesterol metabolism in the liver, as well as inside the KCs, in an established hyperlipidemic low-density lipoprotein receptor knockout (*Ldlr^−/−^*) mouse model. Unlike wildtype mice, the *Ldlr^−/−^* mice demonstrate a human-like lipoprotein profile characterized by mildly elevated cholesterol levels which is mostly carried in the intermediate-density lipoprotein (IDL)/LDL fractions [15]. Additionally, recent research demonstrated that the presence of steatosis and hepatic inflammation is persisted for a long period of time, and even progressed into liver fibrosis [16]. The resemblance with a human-like lipoprotein profile, the sustained hepatic inflammatory response and the development of fibrosis makes hyperlipidemic *Ldlr^−/−^* mice an excellent mouse model to study the onset and progression of NAFLD. We hypothesized that HP-B-CD ameliorates liver cholesterol and intracellular cholesterol levels inside KCs. Once a week, we administered HP-B-CD to *Ldlr^−/−^* mice fed a high-fat, high-cholesterol (HFC) diet. Mice receiving phosphate-buffered saline (PBS) were used as a control. After HP-B-CD treatment, we found that lysosomal cholesterol levels and cholesterol crystallization were decreased inside KCs compared to control-treated hyperlipidemic mice. In contrast, no changes in the total level of liver cholesterol and KC area were seen. These data indicate for the first time that HP-B-CD could be a useful tool to improve intracellular cholesterol levels in the context of the metabolic syndrome.

## 2. Results

### 2.1. No Difference in Liver and Plasma Cholesterol Levels upon HP-B-CD Treatment

**Figure 1:**
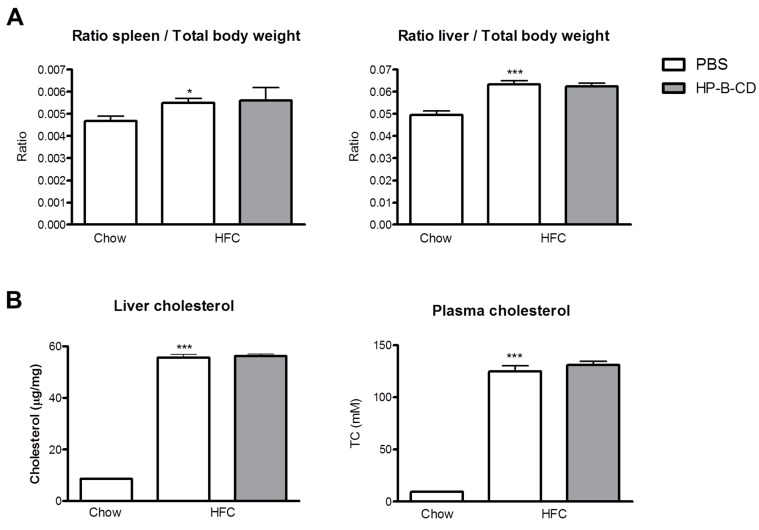
Relative spleen, liver weights and cholesterol levels. (**A**) Relative spleen and liver weights after 12-weeks of regular chow or HFC diet in *Ldlr^−/−^* mice with and without HP-B-CD treatment; and (**B**) Cholesterol levels were analyzed in liver as well as plasma of *Ldlr^−/−^* mice after 12 weeks of regular chow or HFC diet. TC: total cholesterol. Data are expressed as mean ± SEM (*n* = 10 for the chow-fed mice; *n* = 12 for the mice fed an HFC diet without treatment; *n* = 12 for the HFC-fed mice receiving HP-B-CD treatment). * Significantly different from chow. * and *** indicate *p* < 0.05, and 0.001, respectively.

The mean spleen and liver weight in the HFC group was increased compared to chow, but remained similar upon weekly HP-B-CD treatment for a 12-week time period (Figure 1A). In line with these data, liver and plasma cholesterol levels were significantly higher upon HFC feeding than after 12 weeks of regular chow. However, no differences in cholesterol concentrations were found between PBS and HP-B-CD-treated mice on an HFC diet (Figure 1B). Thus, these data indicate that HP-B-CD has no effect on organ weight and cholesterol concentrations in plasma and liver.

### 2.2. Foamy KC Appearance Is Similar between Control- and HP-B-CD-Injected Mice

**Figure 2:**
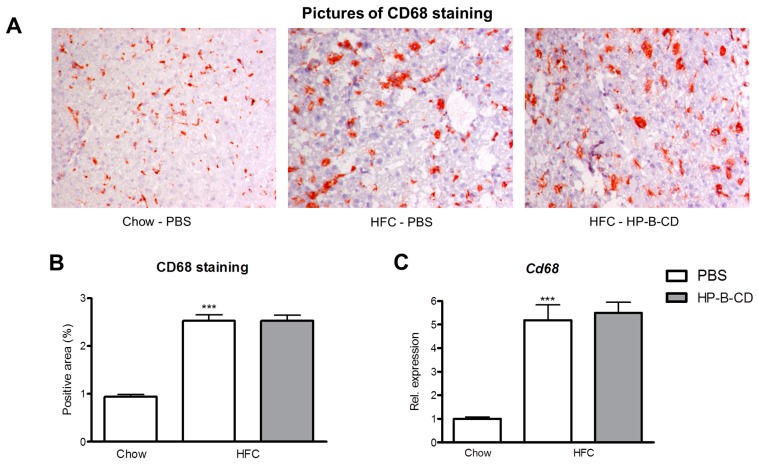
Hepatic CD68 expression. (**A**) Representative histological pictures of the CD68 staining (original magnification, 200×) performed on liver sections of chow, PBS-injected and HP-B-CD-injected HFC-fed mice; (**B**) Quantification of the percentage CD68-positive area; (**C**) Hepatic gene expression analysis of *Cd68*. Gene expression data are shown relative to chow. Data are expressed as mean ± SEM (*n* = 10 for the chow-fed mice; *n* = 12 for the mice fed an HFC diet without treatment; *n* = 12 for the HFC-fed mice receiving HP-B-CD treatment). * Significantly different from chow. *** indicates *p* < 0.001.

To determine whether HP-B-CD affects the foamy appearance of KCs, liver sections were stained against CD68, a marker specifically for macrophages. As expected, HFC feeding increased the area of the KCs, compared to mice fed regular chow. No difference in CD68-positive area was observed between PBS- and HP-B-CD-injected mice on an HFC diet (Figure 2A). These data were confirmed upon quantification of the CD68-positive area of these livers (Figure 2B) and gene expression analysis of *Cd68* (Figure 2C), which both demonstrated no difference in the CD68 expression after HP-B-CD. To summarize, the HFC diet leads to a foamy KC appearance and was not affected upon HP-B-CD treatment.

### 2.3. HP-B-CD-Treated Mice Demonstrate Decreased Lysosomal Cholesterol Accumulation and Cholesterol Crystallization

**Figure 3:**
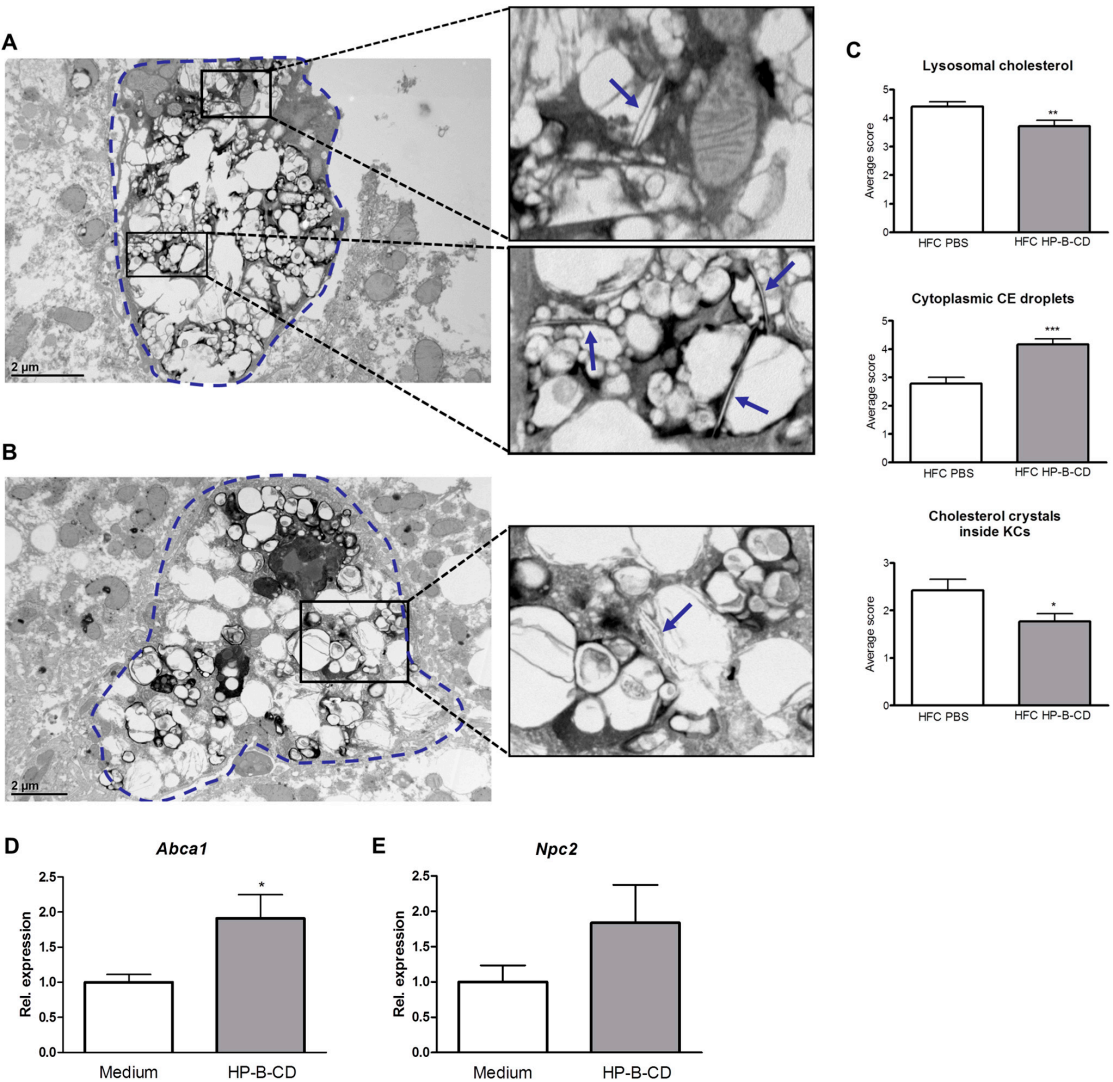
Effect of HP-B-CD on intracellular cholesterol distribution and cholesterol transporters. Representative electron microscopy pictures of Kupffer cells (KCs) of HFC-fed *Ldlr^−/−^* mice without (**A**) and with HP-B-CD treatment (**B**). Lysosomes are indicated in black by ACPase staining. KCs are depicted by the dashed line. Arrows point to cholesterol crystals; (**C**) Scoring of lysosomal cholesterol, cytoplasmic cholesteryl ester (CE) droplets and cholesterol crystals after 12 weeks of HFC diet. In total, 40 to 50 KCs were scored per HFC group and an average score was calculated. Gene expression levels of the cholesterol transporters *Abca1* (**D**) and *Npc2* (**E**) in oxLDL-loaded BMDM with or without HP-B-CD treatment. The *in vitro* results are the mean ± SEM from two separate experiments performed in triplicate. * Significantly different from control. *, ** and *** indicate *p* < 0.05, 0.01 and 0.001, respectively.

Electron microscopy was performed to investigate the effect of HP-B-CD on redirecting lysosomal cholesterol to the cytoplasm and cholesterol crystallization. Livers were fixed and stained for acid phosphatase (ACPase), a marker for lysosomes. As demonstrated in Figure 3A, KCs of the non-treated HFC group displayed increased lysosomal cholesterol accumulation and cholesterol crystals compared to KCs of HP-B-CD-treated mice upon HFC feeding (Figure 3B). In the latter group, cholesterol droplets were mainly observed inside the cytoplasm. Scoring electron microscopy pictures of approximately 50 KCs from both HFC groups confirmed that lysosomal cholesterol was significantly decreased, while cytoplasmic cholesteryl ester (CE) droplets were increased upon HP-B-CD treatment. Moreover, mice administered HP-B-CD had less cholesterol crystals inside their KCs compared to control PBS-injected mice after a 12-week HFC diet (Figure 3C). These results suggest that HP-B-CD is able to redirect cholesterol from the lysosomes to the cytoplasm.

Previous studies found that it is mainly oxidized LDL (oxLDL) that tends to accumulate inside the lysosomes of *Ldlr^−/−^* mice and in cultured macrophages [5,17]. To show that HP-B-CD is able to modify lysosomal oxLDL, we isolated bone marrow-derived macrophages (BMDM) from wildtype mice and stimulated these with oxLDL. Subsequently, BMDM were treated with HP-B-CD (0.3%) or with control medium. Upon HP-B-CD treatment, gene expression of ATP-binding cassette transporter A1 (*Abca1*), a key regulator of cholesterol efflux, was elevated compared to control treatment (Figure 3D). Additionally, the gene expression of Niemann-Pick type C2 (*Npc2*), an intracellular lysosomal cholesterol transporter responsible for cholesterol transport out of the lysosome, was also elevated after HP-B-CD treatment compared to control (Figure 3E). These data demonstrate the ability of HP-B-CD to lower lysosomal oxLDL levels.

### 2.4. Campesterol and 7α-Hydroxycholesterol Are Increased after HP-B-CD Treatment

**Figure 4:**
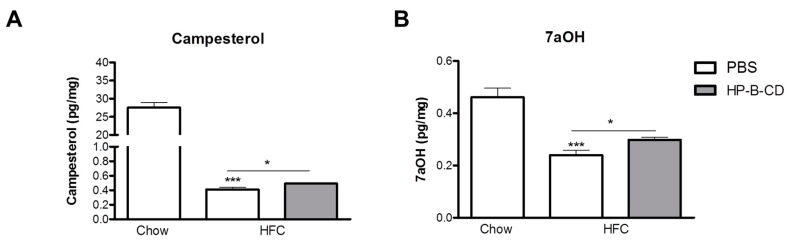
Hepatic levels of campesterol and 7α-hydroxycholesterol (7aOH). Campesterol (**A**) and 7aOH (**B**) were analyzed in liver pieces of *Ldlr^−/−^* mice after 12 weeks of regular chow or HFC diet. Data are expressed as mean ± SEM (*n* = 10 for the chow-fed mice; *n* = 12 for the mice fed an HFC diet without treatment; *n* = 12 for the HFC-fed mice receiving HP-B-CD treatment). * Significantly different from chow. * and *** indicate *p* < 0.05 and 0.001, respectively.

To obtain a better understanding in the cholesterol metabolism after HP-B-CD treatment, we analyzed campesterol, a phytosterol, and 7α-hydroxycholesterol (7aOH), an oxysterol, in the livers of control chow-fed and non-treated and HP-B-CD-treated HFC-fed mice. Hepatic campesterol and 7aOH levels were dramatically reduced upon an HFC diet compared to chow. Interestingly, campesterol and 7aOH were significantly increased after HP-B-CD treatment, although the elevation was minimal (Figure 4A,B).

## 3. Discussion

Currently, no registered therapeutic interventions against NAFLD are available. Previous studies from our group suggest that lysosomal cholesterol accumulation can be considered as a key mechanism for the pathogenesis of NAFLD in mice. As such, we tested HP-B-CD, a compound known to redirect cholesterol from the lysosomes to the cytoplasm in the context of lysosomal storage diseases, to improve the cholesterol metabolism in an established hyperlipidemic mouse model to study NAFLD [16]. Unlike total hepatic cholesterol levels, we now show that it is the intracellular localization of cholesterol in hyperlipidemic mice that is improved after HP-B-CD treatment. Our novel data demonstrate that HP-B-CD reduces lysosomal cholesterol accumulation and cholesterol crystallization in KCs during hyperlipidemic conditions. Moreover, these data underline the shared etiology between lysosomal storage diseases and NAFLD.

Lysosomal cholesterol accumulation could be efficiently overcome by the administration of HP-B-CD to *Npc1^−/−^* mice and cells deficient for the *Npc1* gene [10,13]. In the current study, a similar dosage (20% *w*/*v*, 4000 mg per kg body weight) and product (H107, Sigma-Aldrich) of HP-B-CD was administered subcuteanously as described in previous *in vivo* studies [11,12,13,14,18] and showed to decrease lysosomal cholesterol storage and increase cytoplasmic CE droplets inside KCs. Thus, HP-B-CD was able to reduce lysosomal cholesterol in a lysosomal storage disease and fatty liver disease and suggests a shared disease mechanism. Lysosomal cholesterol accumulation in macrophages is an underlying mechanism in diseases associated with the metabolic syndrome, such as atherosclerosis and NAFLD [4,7,8]. Unlike non-oxidized LDL that accumulates in lysosomes of NPC mice, recent evidence points toward the specific lysosomal trapping of oxLDL in *Ldlr^−/−^* mice and in cultured macrophages [5,17,19,20]. Besides NAFLD, increasing attention has been directed to the crucial role of oxLDL in the pathogenesis of various metabolic diseases, including atherosclerosis [7] and diabetes [21]. However, thus far, oxLDL has been shown to be highly resistant to removal from the lysosome [22] and to intracellular degradation [23]. As such, the ability of HP-B-CD to liberate lysosomal cholesterol in *Ldlr^−/−^* mice is an exciting opportunity for the amelioration of various metabolic diseases underlying lysosomal oxLDL accumulation.

HP-B-CD has cholesterol-binding capacities and normalizes cholesterol homeostasis in *Npc1* deficient cells [24]. Upon absorption, HP-B-CD has been shown to be distributed over several tissues including the liver [25]. In line, numerous studies demonstrated a clear improvement in liver function of *Npc1^−/−^* mice after subcutaneous administration of HP-B-CD [11,12,13,14,26]. Much to our surprise, no changes in plasma and liver cholesterol levels and the foamy KC appearance were found in HP-B-CD-treated mice compared to their control. A possible explanation for these data is that cholesterol storage inside lysosomes is much less extreme in the *Ldlr^−/−^* model compared to the *Npc1^−/−^* mice fed an HFC diet. Therefore, the effect of HP-B-CD on total cholesterol levels, and also liver weight, in the *Ldlr^−/−^* model is not significant. In line with our observations, Taylor *et al.* demonstrated that HP-B-CD treatment does not lead to increased cholesterol levels in urine and plasma, leaving HP-B-CD to liberate lysosomal cholesterol for further processing within the cytosolic compartment only [27]. However, these results may be related to the fact that HP-B-CD was injected only one single time in the latter study.

In line with the unaffected plasma and liver cholesterol levels between HP-B-CD- and control-treated mice, we could not detect any differences in the foamy KC appearance. This is a striking result, since the amount of cholesterol crystals were lowered in mice treated with HP-B-CD compared to PBS and suggest that foamy KCs do not correlate with cholesterol crystallization. This is contrary to the current view that foamy macrophages are strongly associated with cholesterol crystallization [5,28,29,30]. Of note, cholesterol crystallization occurs within lipid-loaded lysosomes and not in the cytoplasm, hereby confirming that the actual formation of cholesterol crystals is dependent on lysosomal cholesterol levels [31]. Indeed, in line with a decreased level of lysosomal cholesterol, we observed less cholesterol crystallization. Altogether, these data indicate that there is dissociation between foam cell formation and cholesterol crystallization.

Despite much effort, the exact mechanism by which HP-B-CD normalizes cholesterol homeostasis is still under debate. After injection, HP-B-CD has the ability to be internalized into the lysosomes of cells via bulk phase endocytosis and release sequestered cholesterol from the lysosome into the cytosol [32]. Due to the unique structure of HP-B-CD, it can serve as a cholesterol sink, extract cholesterol and trap cholesterol in the presence of high cholesterol concentrations. However, during low cholesterol levels, HP-B-CD rather acts as a cholesterol shuttle, transporting cholesterol between membranes. Other evidence points towards HP-B-CD as a compound that extracts cholesterol from cell membranes by which the resulting HP-B-CD-cholesterol complex is then cleared via the kidneys.

In the current study, we have found that the HFC diet leads to a dramatic reduction of liver campesterol compared to chow. In the plasma, campesterol can be considered as a surrogate marker for intestinal cholesterol absorption, and likely has the same function when found in the liver [33]. Intestinal cholesterol absorption, and thus campesterol, is likely to be inhibited during consumption of a high fat diet, as a protective mechanism to prevent excess plasma cholesterol levels. Our data are in line with other studies pointing towards an inverse correlation between campesterol and BMI/obesity [34,35]. Likewise, elimination of overweight by lifestyle interventions normalized intestinal cholesterol absorption [33]. Campesterol and 7aOH in the liver were elevated upon HP-B-CD administration. While the molecular mechanisms behind this observation are not clear, it is known that both campesterol and 7aOH are liver X receptor (LXR) agonists which serve as an intracellular sensor of cholesterol content and mobilize cholesterol to the plasma membrane upon activation [36]. Thus, the upregulation of campesterol and 7aOH levels upon HP-B-CD could possibly contribute to the improved intracellular cholesterol trafficking observed upon administration of HP-B-CD. Despite the upregulation of campesterol and 7aOH upon HP-B-CD treatment, we did not observe a decrease in plasma and liver cholesterol. This observation can be explained by the fact that dietary phytosterols, including campesterol, have been shown to increase the affinity and efficiency of the LDLR for adequate cholesterol removal [37]. Since our study was performed in *Ldlr^−/−^* mice, campesterol was not able to enhance efficiency of the LDLR. Moreover, these results indicate that lysosomal cholesterol levels were reduced independent of the LDLR and support the view of campesterol and 7aOH being an LXR-agonist. Thus, campesterol and 7aOH levels were upregulated upon HP-B-CD and possibly improved intracellular cholesterol trafficking via LXR signaling.

## 4. Experimental Section

### 4.1. Mice, Diet and Injections

The mice were housed under standard conditions and given free access to food and water. All experiments were approved by the Committee for Animal Welfare of Maastricht University and performed according to Dutch regulations. Eleven to twelve-week old female *Ldlr^−/−^* mice on a C57/Bl6 background were either fed regular chow (*n* = 10) or an HFC diet (*n* = 12 per HFC group with and without HP-B-CD treatment) for 12 weeks. The effects of HP-B-CD were investigated by giving weekly subcuteanous injections at the start of the HFC diet with 4000 mg per kg of body weight of 20% *w*/*v* HP-B-CD (H107, Sigma-Aldrich GmbH, St. Louis, MO, USA) (*n* = 12). PBS was used for control injections. The HFC diet contained 21% milk butter, 0.2% cholesterol, 46% carbohydrates and 17% casein. Collection of blood and tissue specimens, biochemical determination of lipids in plasma, liver histology, electron microscopy, acid phosphatase (ACPase) enzyme cytochemistry, RNA isolation, complementary DNA synthesis and quantitative polymerase chain reaction were determined as described previously [4,5,38,39,40,41]. Pieces of liver were used for quantification of liver cholesterol and the hepatic levels of campesterol and 7α-hydroxycholesterol as described previously [42].

### 4.2. CD68 Staining

For the CD68 staining, six microscopical views (200× magnification) of each liver were obtained. Adobe Photoshop CS2 v.9.0 was used to analyze CD68-positive (red) pixels as well as total unstained tissue pixels of each microscopical picture. Subsequently, these data were used to calculate the percentage of CD68-positive area.

### 4.3. Scoring of Lysosomal Lipid Droplets, Cytoplasmic CE Droplets and Cholesterol Crystals

Electron microscopy was performed by an expert in the electron microscopical field of the liver. By using electron microscopy pictures, analysis of lysosomal cholesterol was performed by scoring the area of lysosomal lipid droplets, those that are inside ACPase-positive lysosomes indicated by the black membrane, and the area of cytoplasmic CE droplets in 40 to 50 KCs from each HFC group. Each KC was scored between 0 and 6; 0 indicated no lipid droplets inside lysosomes or no cytoplasmic CE droplets, whereas an extremely large area of lysosomal lipid droplets or cytoplasmic CE droplets was scored with a 6. Subsequently, the average lysosomal cholesterol and cytoplasmic CE area per KC was calculated. The scoring and the average calculation for cholesterol crystallization were performed similarly; the score 0 indicated no cholesterol crystals, while 5 indicated the highest area of cholesterol crystals and was performed as described previously [29].

### 4.4. Bone Marrow-Derived Macrophages

Bone marrow-derived macrophages (BMDM) were isolated from the tibiae and femurs of wildtype C57BL/6 mice. Cells were cultured in RPMI-1640 (GIBCO Invitrogen, Breda, The Netherlands) with 10% heat-inactivated fetal calf serum (Bodinco B.V. Alkmaar, The Netherlands), penicillin (100 U/mL), streptomycin (100 μg/mL) and l-glutamine 2 mM (all GIBCO Invitrogen), supplemented with 20% L929-conditioned medium (LCM) for 8–9 days to generate BMDM. After attachment, macrophages were seeded at 350,000 cells per well in 24-well plates and incubated for 72 h with oxLDL (25 μg/mL; Alfa Aesar: J65591, Wardhill, MA, USA), followed by a treatment with or without 0.3% HP-B-CD (H107, Sigma-Aldrich GmbH, St. Louis, MO, USA). Then cells were washed and stimulated with lipopolysaccharide (100 ng/mL) for 4 h. Finally, cells were lysed and further processed for gene expression analysis.

### 4.5. Statistical Analysis

Data were analyzed by two-tailed, unpaired, *t*-tests using GraphPad Prism, version 4.0 for Windows (GraphPad Software Inc., La Jolla, CA, USA). Data are represented as mean ± standard error of mean (SEM) and considered significant at *p* < 0.05 (* *p* < 0.05; ** *p* < 0.01 and *** *p* < 0.001, respectively).

## 5. Conclusions

Unlike total liver cholesterol, administration of HP-B-CD improves intracellular cholesterol localization inside KCs of NAFLD-susceptible *Ldlr^−/−^* mice. Therefore, HP-B-CD could be a useful tool to improve intracellular cholesterol levels and cholesterol crystals in the context of the metabolic syndrome and should be tested in the future. Further studies are necessary to determine the novel role of oxysterols and phytosterols in improving intracellular cholesterol trafficking. Additionally, these data underline the existence of a shared etiology between lysosomal storage diseases and NAFLD.

## Abbreviations

NAFLD: Non-alcoholic fatty liver disease; KCs: Kupffer cells; NPC: Niemann-Pick Type C; HP-B-CD: Two-hydroxypropyl-β-cyclodextrin; LDL(R): Low-density lipoprotein (receptor); IDL: Intermediate-density lipoprotein; HFC: High-fat, high-cholesterol; PBS: Phosphate-buffered saline; CE: Cholesteryl ester; ACPase: Acid phosphatase; 7aOH: 7α-Hydroxycholesterol; OxLDL: Oxidized LDL; LXR: Liver X receptor.
